# Effects of Different Mineral Admixtures on the Properties of Fresh Concrete

**DOI:** 10.1155/2014/986567

**Published:** 2014-02-18

**Authors:** Sadaqat Ullah Khan, Muhammad Fadhil Nuruddin, Tehmina Ayub, Nasir Shafiq

**Affiliations:** Civil Engineering Department, Universiti Teknologi PETRONAS, Block 13, Level III, 31750 Tronoh, Perak, Malaysia

## Abstract

This paper presents a review of the properties of fresh concrete including workability, heat of hydration, setting time, bleeding, and reactivity by using mineral admixtures fly ash (*FA*), silica fume (*SF*), ground granulated blast furnace slag (*GGBS*), metakaolin (*MK*), and rice husk ash (*RHA*). Comparison of normal and high-strength concrete in which cement has been partially supplemented by mineral admixture has been considered. It has been concluded that mineral admixtures may be categorized into two groups: chemically active mineral admixtures and microfiller mineral admixtures. Chemically active mineral admixtures decrease workability and setting time of concrete but increase the heat of hydration and reactivity. On the other hand, microfiller mineral admixtures increase workability and setting time of concrete but decrease the heat of hydration and reactivity. In general, small particle size and higher specific surface area of mineral admixture are favourable to produce highly dense and impermeable concrete; however, they cause low workability and demand more water which may be offset by adding effective superplasticizer.

## 1. Introduction

Admixtures are added in concrete to improve the quality of concrete. Mineral admixtures include fly ash (*FA*), silica fume (*SF*), ground granulated blast furnace slag (*GGBS*), metakaolin (*MK*), and rice husk ash (*RHA*) which possess certain characteristics through which they influence the properties of concrete differently. The reported benefits of mineral admixtures are often associated with the harden properties of concrete; however, mineral admixtures may also influence the properties of wet concrete between the time of mixing and hardening in one or more of the following ways such as they may affect water demand, heat of hydration, setting time, bleeding, and reactivity. In the authors' view, there is no literature that summarizes the effect of these mineral admixtures on the properties of fresh concrete. Moreover, effect of mineral admixtures on the durability and on the mechanical properties of concrete remained a focus of interest. Nevertheless, effect of mineral admixtures on the properties of fresh concrete is very important as these properties may affect the durability and mechanical properties of concrete. Comparative studies have been done such as the effect of blast furnace slag and fly ash on the hydration of fresh cement paste [1], effect of silica fume (*SF*), metakaolin (*MK*), fly ash (*FA*), and ground granulated blast furnace slag (*GGBS*) on the setting times of high-strength concrete [2]. This paper has been written to summarize the available literature and provide the reader with a distinctive comparative analysis on the effect of mineral admixtures on water demand, heat of hydration, setting time, bleeding, and reactivity of concrete. Also the effect of physical and chemical characteristics of fly ash (*FA*), silica fume (*SF*), ground granulated blast furnace slag (*GGBS*), metakaolin (*MK*), and rice husk ash (*RHA*) on the fresh concrete has been reviewed.

## 2. Background

McMillan and powers were the first who used coal *FA* in concrete in 1934 [3]. After them, based on the research work conducted during the 1950s by Fulton and Marshall [4], Lednock, Clatworthy and Lubreoch Dams had been constructed in the UK using *FA* as a partial cementitious material, and since then, these structures have been reported in excellent conditions [5]. *FA* or Pulverized-fuel ash (*PFA*) from coal is a pozzolan, which results in low-permeable and more durable concrete, which is able to resist the ingress of deleterious chemicals. This pozzolan was first reported as a controller of damaging alkali-silica reaction (ASR) in 1949 by Blanks [6]. Alkalis available in *FA* concrete are usually higher than the concrete without *FA* which confirms that *FA* plays a preventive role against ASR that is an added advantage of *FA* [3]. Two dams Nant-y-Moch Dam (central Wales, UK) and the Lower Notch Dam (Ontario, Canada) were constructed in 1962 and 1969 with *FA* concrete. These structures were inspected in 1991 after about 35–40 years of their construction. The conditional survey, petrographic examination, and damage rating index (DRI) showed no symptoms of ASR [7]. In 2010, these structures were once again visually inspected and it was reported that both dams are in excellent condition after 40 to 50 years in service. This report indicates a strong evidence of the benefits of using *FA* [3].

In 1862, *GGBS* was first discovered in Germany by Emil Langen; however, commercial production of lime-activated *GGBS* was started in 1865 in Germany [5]. Concrete containing *GGBS* has been named in the literature as slag concrete, which is being used successfully in many countries due to accepted benefits of this material and the use of slag concrete has been recommended in their national standards [5]. Around 1880, *GGBS *was first used with Portland cement (*PC*). Since then it has been used extensively in many European countries. In the UK, the first British standard for Portland blast furnace cement (*PBFC*) was introduced in 1923 [5].

In 1947, *SF *was first obtained in Norway, during filtration of the exhaust gases from furnaces as fumes. The large portion of these fumes contained very fine powder of high percentage of silicon dioxide. Since the 1970s, filtration of gases has started at large scale and, in 1976, first standard NS 3050 was granted to use *SF* in factory-produced cement, extensive literature is available on *SF* and *SF* concrete [5]. It is a high quality material used in the cement and concrete industry. It has been reported that if a typical dosage of *SF* of 8–10% by weight of cement is added in concrete, then its effect is between 50,000 and 100,000 microspheres percent particle; that is, concrete mix will be denser and cohesive due to fine particles of *SF* [3].

*MK* is a processed amorphous silica material and it is obtained from calcination of kaolin to a temperature between 600 and 850°C (1112 to 1562°F) [8–11]. Kaolin is naturally occurring material; the chemical and mineralogical compositions are highly dependent on the rock from which it is formed [12]. Kaolin is widely occurring white clay resulting from natural decomposition of feldspar and is mainly used in the manufacturing of porcelain, as a filler in the paper and textiles, and as absorbent in medicines [5]. Saad et al., 1982 [13]; Larbi, 1991 [14]; Halliwell, 1992 [15]; and Hewlett, 2004 [16] reported that the inclusion of *MK* in concrete has no drawbacks; however, in 1995, Martin [17] reported that the inclusion of *MK* in concrete increases the compressive strength up to 110 MPa (16 ksi) with superplasticizer to overcome the higher water requirement in *MK* concrete. In 1996, Wild et al. [18] found that the optimum OPC replacement with *MK* is 20% by weight with superplasticizer of 2.4% of binder weight. Recently, Duan et al., 2013 [19], studied the pore structure and interfacial transition zone (ITZ) of concrete using *GGBS*, *SF,* and *MK* and it has been found that *MK* has positive effects on pore structure and ITZ enhancement of concrete higher than *SF* and *GGBS* [19].

Rice husk is the outer skin of the rice grain with a high concentration of silica, generally more than 80–85% [20]. It occupies about 30% of the gross weight of a rice kernel and normally contains 80% of organic and 20% of inorganic substances [21]. *RHA* has high pozzolanic activity due to noncrystalline silica and high specific surface area. *RHA* has been used in lime pozzolan mixes for partly replacement of Portland cement [22–27].

## 3. Manufacturing

*FA *is produced when coal is burnt during power generation about 1600°C (2912°F) [28]. This burning also results in some incombustible materials which amalgamate to form spherical glassy droplets of silica (SiO_2_), alumina (Al_2_O_3_), iron oxide (Fe_2_O_3_), and other minor constituents. According to ASTM C 618-05, there are two classes of *FA* based on the types of coal from which it originates. The Class F is produced by burning anthracites which is mainly a siliceous and possesses pozzolanic characteristics. The Class C contains lime and higher MgO content and it is produced by burning lignite and subbituminous coal. Class C fly ash is lighter in colour in comparison with other ashes and may cause expansion and their strength behaviour at high temperature is not apparent [29, 30].

*GGBS* contains silicates and alumino-silicates of calcium and is a by-product of iron manufactured in a blast furnace. There are two techniques, granulation and pelletization, through which *GGBS* is produced. In granulation technique, molten slag is forced over a weir into high pressure water jets which rapidly cools the slag as granules of 5 mm (0.197 in.) diameter; however, in pelletization technique, molten slag is poured into cold water rotating drum. The fins inside the rotating drum throw molten slag in the air towards the walls of the drum where water is sprayed to cool it rapidly. This process produces particles of size 100 mm (3.94 in.) to dust, out of which particle size of less than 6 mm (0.236 in.) in size is used to manufacture the *GGBS*. The materials produced from these two techniques can be used as raw material for glass structured *GGBS *[5].

*SF* is a by-product obtained after reducing high-purity quartz with coal in electric arc furnace by heating up to 2000°C (3632°F) during the production of silicon. By oxidation and condensation of exhaust gas SiO, very fine spherical particles of *SF* are obtained which are highly reactive with the Ca(OH)_2_ produced during hydration of cement [5]. *SF *consists of extremely fine particles of average diameters 0.1 *μ*m (0.00000394 in.), having pozzolanic characteristics and tendency to develop plastic shrinkage cracks [29, 30].

Kaolin is converted into *MK *when it is heated to the temperature between 600 and 850°C (1112 to 1562°F) [8–11]. *MK* is a very reactive pozzolan, but its physical and chemical characteristics greatly depend on the raw material used, the temperature during calcination and finishing process (rapid cooling after calcination) [5]; however, *MK* with a highly disorganized structure has been produced by normal cooling as well [8–11]. The temperature of calcination and duration depended on the mineralogical composition of raw kaolin. It has been reported that higher alunite content in kaolin requires higher temperature of calcination and low alunite content gives good calcined kaolin on low temperature [10, 11].

Rice husk is produced in millions of tons per year as a waste material in agricultural and industrial processes. *RHA* is produced by slow burning of rice husk at a temperature between 500 and 700°C (932 to 1292°F) [30]. *RHA* may produce about 20% by weight of rice husk after incineration [21].

## 4. Chemical Reaction of Mineral Admixtures with Ordinary Portland Cement

*FA* reaction with ordinary Portland cement (OPC) is a two-stage process. In the first stage, during the early curing, the primary reaction is with CaOH_2_; however, the reaction rate depends on the curing temperature. At room temperature, the slower CaOH_2_ activation minimizes the reaction rate. The effectiveness of the use of fly ash in concrete depends on many factors including the following:

- the chemical and phase composition of *FA* and OPC;
- Ca(OH)_2_ concentration of the reaction system;
- the morphology of *FA* particles;
- the fineness of *FA* and OPC;
- the development of heat during the early age of hydration process;
- the reduction in mixing water requirements with *FA*.

Variations in chemical composition and reactivity of *FA* affect early stage properties and the rheology of concrete [28]. It is advised to determine the acceptability of *FA* through trial mixes by considering workability, strength development, and durability [28]. In the second stage, with a continuing supply of moisture, the lime reacts pozzolanically with the *FA* and produces additional hydration products of a fine pore structure.

The pozzolanic reaction may be represented as

(1) Calcium  hydroxide+silica=tricalcium  silicate+water3Ca(OH)2+SiO2=3CaO·SiO2+3H2O

Cabrera and Plowman (1987) showed that calcium hydroxide (lime) depletes with time and its reaction affects the long-term gain of strength in *FA* concrete as compared to ordinary Portland cement concrete; however, despite this reduction, there is sufficient lime to maintain a high pH. It is important to mention that the resulting products due to the addition of *FA* are different from the resultant products formed in OPC concrete. *FA* produces very much finer pore structure with time presuming there is reached to water to maintain the hydration process [5]. A pH more than 13 at 20°C (68°F) is required with the lime to start the reaction by decomposing the Si–O–Si link in *FA *[31].

Unlike *FA* in which Si–O–Si link has to break to make it reactive with lime, *GGBS* requires to be activated to react with lime. *GGBS* due to its glassy structure reacts very slowly with water in the presence of activators. Commonly, sulphates and/or alkalis act as activators, reacting chemically with the *GGBS*. These activators disturb the glassy structure and react to increase the pH of the system up to critical. In contrast to *FA*, *GGBS* only needs a pH level less than 12 and activators. In concrete, due to hydration of cement, Ca(OH)_2_ is produced and acts as an activator [31].

*SF* as a pozzolan reacts with Ca(OH)_2_ and about 25% of *SF* can consume most of the Ca(OH)_2_ at 28 days. This is very important as the Ca(OH)_2_ crystals are relatively weak, brittle, and not cementitious and cracks can easily propagate through regions concentrated with Ca(OH)_2_ crystals, that is, the aggregate cement paste matrix interface [32].

The calcination process (dehydroxylation) of kaolin is actually a transformation from crystalline to amorphous phase. The amount and type of amorphous phase influence the activity of the additives [16]. There are two properties that comprise the activity of additives: chemical activity (usually pozzolanic) and microfiller effect. The former is sturdily linked to the crystallinity of the source kaolin [8]; that is, well structured kaolinite is changed into less reactive *MK *[33]. Dehydroxylation of kaolinite at atmospheric condition costs mass loss of 13.76%, and in result changes SiO_2_·2Al_2_O_3_·2H_2_O into SiO_2_·2Al_2_O_3_ [8]. It is also reported that, after dehydroxylation at 570°C (1058°F), kaolin completely changes to amorphous phase and the chemical activity is a linear function of amorphous phase content in its range of 50–100% [8]. *MK* of amorphous phase content less than 20% can be considered as inert materials in pozzolanic activity viewpoint [8]. Amorphous phase contents in *MK* have also influenced the activity strength index (the ratio of the compressive strength of standard mortar cubes, prepared with 80% reference cement plus 20% additive by mass, to the compressive strength of the standard mortar cube prepared with reference cement only, tested at the same age); however, there is no increase in activity strength index by the increase of amorphous phase index above 55% [8].

Yu et al., 1999 [34], found that the reaction between *RHA* and Ca(OH)_2_ solution yields C–S–H gel. The morphology of C–S–H gel is like congregate, having a large specific area due to the higher porous structure. This C–S–H gel and its large specific area are the main reason improving the concrete properties with the addition of *RHA *[34, 35].

## 5. Physical and Chemical Characteristics of Mineral Admixtures 

The physical and chemical properties of mineral admixtures and OPC have been presented in Table 1. Though these physical and chemical properties are generally varied depending on the source from which these mineral admixtures are derived, the variation in these properties seldom too large and comprehensive comparison is possible through these properties. The first property is the specific gravity. The mineral admixtures presented in Table 1 have lesser specific gravity than OPC. Therefore, more volume is expected when any one of these mineral admixtures replaces OPC by mass. Generally reduction in fine aggregate contents is necessary to overcome the volume increase.

The most important constituents for any mineral admixture are silica and alumina oxides. In comparison with OPC, the mineral admixtures presented in Table 1 have higher quantity of silica oxide in their constituent. The maximum content of silica oxide is in *RHA* and *SF* showing their reaction capability with the primary hydrate of cement to produce calcium silicate hydrate (CSH) which is strengthening gel in concrete; however, the content of alumina oxide is lesser in *SF* and *RHA*. On the other hand, *MK* has substantial contents of silica and alumina oxide showing its capability to produce calcium silicate hydrate (CSH) and calcium aluminate hydrate (CAH) which has also bonding characteristics in the concrete.

The particle size and surface area are the utmost important for mineral admixtures. As reported in the literature, smaller particle size with greater surface area is favourable within concrete to be more reactive with the alkaline environment [3]. In Table 1, *RHA, SF, *and* MK *have the smaller particle size and greater surface area showing their capability to react more effectively with Ca(OH)_2_ in the concrete.

**Figure 1:**
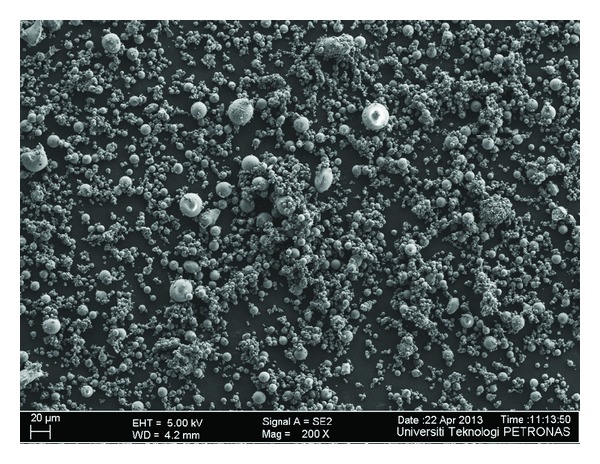
Microstructure of fly ash through field emission scanning electron microscope (FESEM) showing particle size distribution.

**Figure 2:**
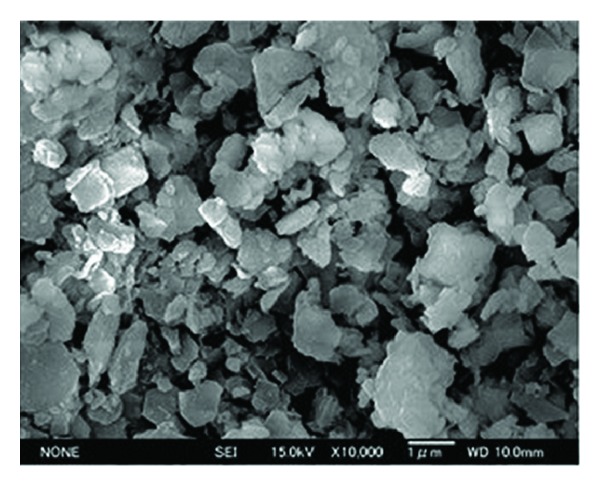
Microstructure of blast furnace slag through field emission scanning electron microscope (FESEM) showing particle size distribution [36].

**Figure 3:**
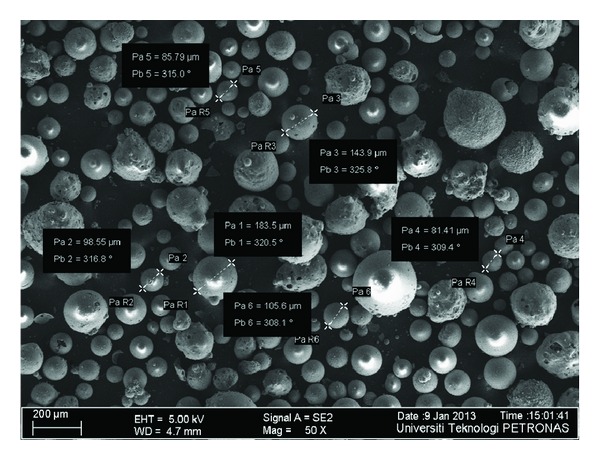
Microstructure of silica fume through field emission scanning electron microscope (FESEM) showing particle size distribution.

**Figure 4:**
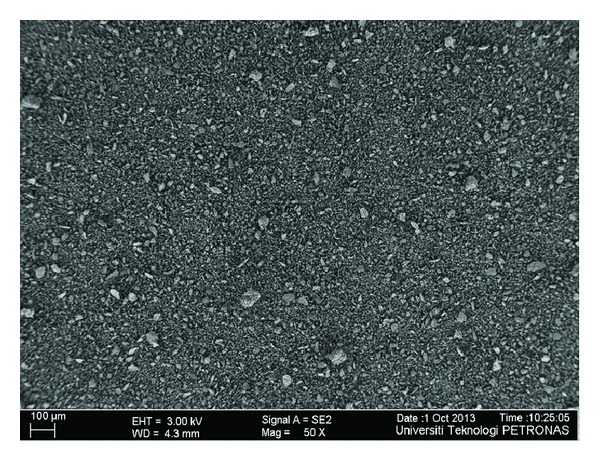
Microstructure of metakaolin through field emission scanning electron microscope (FESEM) showing particle size distribution.

**Figure 5:**
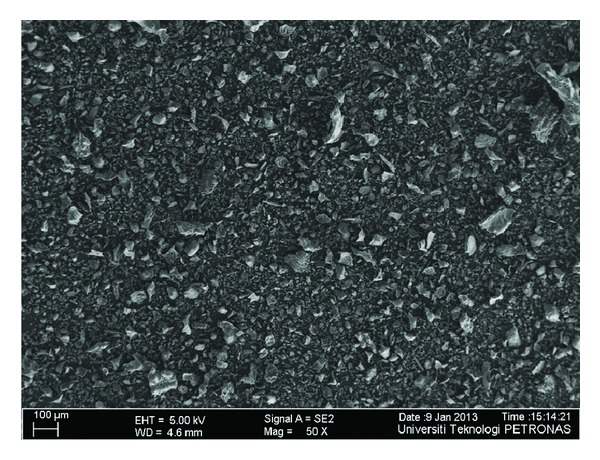
Microstructure of rice husk ash through field emission scanning electron microscope (FESEM) showing particle size distribution.

The microstructure of *FA*, *GGBS*, *SF*, *MK,* and *RHA* through field emission scanning electron microscope (FESEM) has been shown in Figures 1, 2, 3, 4, and 5. Apparently *GGBS* and *RHA* have most uneven particle size distribution even though these FESEM images are taken after grinding. Hence, it may infer that grinding procedure and duration are very important in case of *GGBS* and *RHA*. *GGBS*, *MK*, and *RHA* have particles of irregular shape having multiple layer structure. Whereas, the* FA *and *SF *have spherical shape particles showing a possible increase in flow ability by packing of material once used in the concrete.

## 6. Properties of Fresh Concrete

### 6.1. Water Demand or Workability

According to Owens, 1979 [37], strength performance of a single source/type of *FA* can be related to water demand and fineness. For a specific workability, water demand can be reduced by varying particle shape and using finer fractions of *FA *[37]. Dewar, 1986 [38], correlated the mix design system with water reductions using *FA* and found that the higher strength of *FA* concrete depends on reducing the water content and pozzolanic performance of the cement/*FA* combination. It is generally believed that finer *FA* significantly improves strength with time [39]. Monk, 1983, concluded that, with relatively coarse *FA* of 45 *μ*m (0.0018 in.) residue > 12 percent, the water requirements are greatly reduced [40]. The fluidity of fresh concrete is increased due to spherical particles of *FA*.

Similar to *FA*, less water content is required when *GGBS* is added. Generally, 25% to 70% of cement is replaced with *GGBS* in the concrete [41]. *GGBS* requires about 3% lesser water content in comparison to OPC for the equal slump requirement. This is due to the smooth surface texture of the slag particles that delay the chemical reaction and increase the setting time [5].

*SF* as cement replacing by mass increased the cohesiveness of concrete and required higher water content to maintain workability; however, through vibration or pumping, *SF *due to spherical particles gives concrete greater flow ability as compared to ordinary concrete [5]. The cohesiveness is also due to high early reactivity and lower setting time. Mostly, plasticizer or superplasticizer is used in order to manufacture *SF* concrete without increasing the water/binder ratio, as it is important to use *SF* without strength loss. As a thumb rule, one part of *SF* can replace 3 to 4 parts of the cement by mass without loss of strength, provided the water content remains constant [42].

*MK *increases the cohesiveness of concrete [5, 43]. The requirement of water may be offset by adding plasticizer [5]. The reason for higher water demand in the case of *SF* and *MK* is that both mineral admixtures have high reactivity and consume water very early.

The superplasticizer content needs to be increased if finer and higher percentage of *RHA* has to be used; this is also due to the high specific area of finer *RHA* which increases the water demand. The superplasticizer content has to increase more than 2.0% for *RHA* having a particle size of 11.5 *μ*m (0.00045 in.) and specific surface of 30.4 m^2^/g (148533.6 ft^2^/lb.) [44].

**Figure 6:**
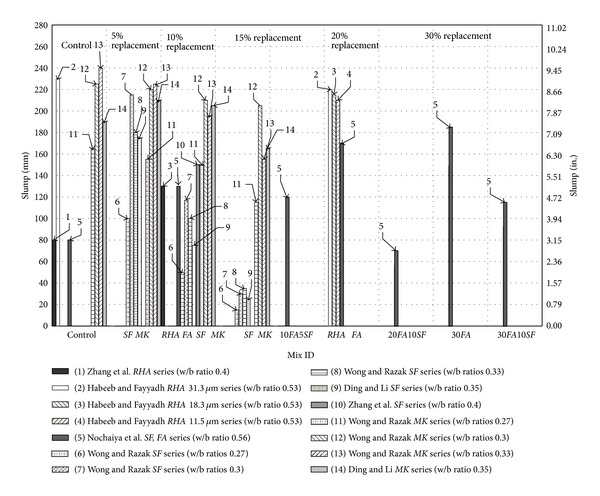
Slump of concrete with mineral admixtures.

In Figure 6, the slump of concretes incorporating mineral admixtures with different water/binder ratio has been plotted. The values of slump have been obtained from the literature. Five experimental investigations have been chosen to summarize the effect of different replacement levels of *FA*, *SF*, *MK,* and *RHA* with different water/binder ratios. Zhang and Malhotra, 1996 [24], investigated *RHA* and *SF* as cement replacing material. From their experimental results, the slumps of concrete having water/binder ratio 0.4, maximum binder content 386 kg/m^3^ (24 lb/ft^3^), and 10% cement replacement have been taken [24]. Habeeb and Fayyadh, 2009 [44], investigated *RHA* of different particle size (fineness) as cement replacing material. From their experimental results, the slumps of concrete having water/binder ratio 0.53, maximum binder content 391 kg/m^3^ (24.4 lb/ft^3^), and 20% cement replacement have been used [44]. Nochaiya et al., 2010 [45], investigated *FA* and *SF* as cement replacing material. From their experimental results, the slumps of concrete having water/binder ratio 0.56, maximum binder content 360 kg/m^3^ (22.45 lb/ft^3^), and 10, 15, 20, 30, and 40% cement replacement with the* FA* or a combination of *FA* and *SF* have been plotted [45]. Wong and Razak, 2005 [46], investigated *SF* and *MK* as cement replacing material. From their experimental results, the slumps of concrete having water/binder ratio 0.23, 0.3 and 0.33; maximum binder content 500 kg/m^3^ (31.18 lb/ft^3^); and 5, 10, and 15% cement replacement with *SF* and *MK* have been shown [45]. Ding and Li, 2002 [47], also investigated *SF* and *MK* as cement replacing material with fixed water/binder ratio of 0.35. From their experimental results, the slumps of concrete having maximum binder content 462 kg/m^3^ (28.82 lb/ft^3^) and 5, 10, and 15% cement replacement with *SF* and *MK* have been used [45].

From Figure 6, there are several important aspects of concrete that can be identified with different mineral admixtures. First is the replacement level. The content of *SF* and *MK* is 15% maximum, whereas *FA* and *RHA* have 30% and 20% maximum replacement levels, respectively. With respect to water/binder ratio, at 5% replacement level, the maximum slump is with *MK* concrete having water/binder ratio 0.33 (Wong and Razak series). Similarly, the maximum slump of *SF* concrete is with the water/binder ratio of 0.3 (Wong and Razak series). Further increase in water/binder ratio caused reduction in slump. Moreover, *MK* concrete has better slump as compared to *SF* concrete as shown in Figure 6. At 10% replacement level the maximum slump is again with *MK* concrete but with water/binder ratio 0.3 (Wong and Razak series). The maximum slump of *SF* concrete at this replacement level is with water/binder ratio of 0.4 (Zhang et al. series). At this replacement level, *FA* and *RHA* concretes have the same slump having water/binder ratio of 0.4 (Zhang et al. series) and 0.56 (Nochaiya et al. series), respectively. At 15% replacement level the maximum slump is again with *MK* concrete but with water/binder ratio 0.3 (Wong and Razak series). The slump of *SF *concrete at this replacement level is extraordinarily reduced. It is also evident from Figure 6 that, with the increase in replacement level, the slump of *MK *concrete is less affected; however, *SF *concrete is very susceptible with the increase in replacement level specifically from 10% to 15%. The slump of concrete having combination of *FA* and *SF* as cement replacement has also been influenced by the addition of *SF* and the slump is further reduced with the increase in *SF* content. Though increase in *FA* content increase the slump as shown in Figure 6, affects of *SF* on the slump are not fully canceled by *FA*. From Figure 6, it is also evident that increase in fineness of *RHA* caused reduction in slump (Habeeb and Fayyadh series) [44]. In general, water demand greatly depends on the particle size, specific surface area, particle shape, replacement level, and reactivity of particular mineral admixture used in concrete. In general, smaller the particle size and higher the specific surface of mineral admixture increases the water demands of concrete. Details of particle size and specific surface of mineral admixtures have been mentioned in Table 1. By comparing the particle size and specific surface of mineral admixtures and available literature, it may propose that *SF *and *MK* require more water content due to small particle sizes, higher specific surface area and high reactivity.

### 6.2. Heat of Hydration

According to Woolley and Conlin, 1989 [54], and Keck and Riggs, 1997 [55], use of *FA* is effective in reducing heat of hydration. The exothermic reaction during the hydration of OPC liberates energy of 500 J/g (10455.8 lb. ft./oz). Use of *FA* by replacing some content of cement influences temperature rise during hydration process and increases pozzolanic reaction with increasing temperature; however, the peak temperature in *FA* concrete is significantly lower than equivalent ordinary Portland cement concrete.

Similar to *FA*, as the proportion of *GGBS* is increased, the heat of hydration is reduced. This is beneficial in large concrete pouring that enable reduced temperature rise which will reduce the probability of thermal cracking [5]. Cheng-yi and Feldman, 1985 [56], have studied heat of hydration of cement pastes containing 0, 10, 20, and 30 percent *SF *by using conduction calorimetry. By increasing the content of *SF*, higher heat of hydration has been observed. The rate of heat liberation, expressed on a cement basis, is increased as the amount of *SF *increases, but the total heat liberated, expressed on a total solid basis in the mixture, somewhat is decreased as *SF* is substituted for cement [42].

Ambroise et al., 1994 [57], pointed the temperature rise of *MK* mortars relative to the plain OPC mortar showing an accelerating effect of *MK* on OPC hydration. Note that the maximum observed temperature rise occurs at 10% replacement of OPC by *MK*. This temperature rise has also been confirmed by Zhang and Malhotra, 1995 [58]. The high reactivity of *MK* with Ca(OH)_2_ is the cause of increase in temperature.

Similar to *FA* and *GGBS*, partial replacement of cement with *RHA* in concrete slows down the rate of hydration in comparison with ordinary concrete. The rate of hydration remains slow during the initial three days and consequently affects the strength after 150 days [59]. In general, it is well evident that finely divided highly reactive pozzolan reacts with Ca(OH)_2_ supply to early heat evolution, by accelerating the hydration of OPC [56, 57] and by rapidly reacting with Ca(OH)_2_ [18].

### 6.3. Setting Time

The setting time of concrete in the context of *FA* depends on ambient temperature, cement content, fineness, water content, dosages of chemical admixtures, content of *FA*, fineness, and chemical composition of *FA* [28]. Tests of concrete containing Class F and Class C *FA* from 10 different sources have been conducted to estimate water requirement, setting time, and bleeding [60]. The water requirement was reduced for concretes with Class C *FA*. No steady water reduction was observed in Class F *FA* concrete. Minor increase in setting time was observed in *FA* concretes [60].

Since *GGBS* slowly reacts with water as compared to Portland cement, therefore stiffening/setting time of concrete is high. The setting time will be greater at high replacement levels above 50% and at lower temperatures (below 10°C (50°F)) [5]. *SF*, having a greater surface area and higher silicon dioxide content, is found to be more reactive than Pulverized fly ash (*PFA*) or *GGBS*. The high reactivity increases hydration rate of the C3S fraction of the cement in the first instance and thus decreases the setting time [5].

Initial and final setting times of concrete containing 10% *MK* are increased and are extended further with the increase in replacement level; however, by increasing the replacement level up to 15%, minor drop in initial setting time particularly is observed in comparison with 10% *MK* concrete [61]. This might be due to the greater water demand of *MK* at the higher replacement level. With higher *MK* content, concrete due to higher binder phase becomes denser and results in accelerating the setting [61]; however, use of effective superplasticizer offsets the high water demand and low water content and ultimately increases the setting time [61].

In comparison to *SF* concrete, the initial and final setting time of *RHA* concrete has been observed and initial and final setting time of *RHA* concrete is about 29 and 60 minutes longer than *SF* concrete, respectively [24]. Hence, the comparison of various admixtures showed that there is a difference in the setting time of *SF* and *MK* concrete as compared to plain concrete. This may be due to the fact that *SF* and *MK* accelerate the hydration of cement after initial set [61]. In general, water content, initial and curing temperature, dosage, source and type of mineral admixtures and superplasticizer, and composition of cement influence the setting time of concrete [61]. Thus, on similar conditions, increase in replacement levels of mineral admixtures in concrete causes decrease in setting time [61].

### 6.4. Bleeding

Gebler and Klieger, 1986 [60], reported that concrete with *FA* showed less bleeding than plain concrete. Also, concrete with Class C *FA* showed lesser bleeding than concretes with Class F *FA*. The reduction in bleeding is due to greater surface area of particles of fly ash and lower water content with fly ash for a given workability [28]. On the contrary, concrete containing *SF* or *RHA *produces no bleeding water [24]. Similarly, the use of *MK* as a partial replacement of cement in suitably designed concrete mixes improves cohesion and reduces bleeding of fresh concrete [5].

### 6.5. Reactivity

Reactivity of pozzolan may be compared with the help of Chapelle test. This test is performed by the reaction of calcium hydroxide with the dilute slurry of the pozzolan at a temperature of 95°C (203°F) for 18 hours. After reaction, the consumed calcium hydroxide is determined. Largent, 1978 [53], reported the results and showed that the reactivity of *MK* is higher than the other pozzolan. The authors conducted the same test on *SF* and *MK* as shown in Table 1 and verified the results reported by Largent, 1978. Larbi, 1991 [14], demonstrated that *MK* in a cement matrix eliminated the calcium hydroxide completely. Nevertheless, *MK* reduces the calcium hydroxide level in concrete but pH remains stable above 12.5 [62].

## 7. Conclusions

Based on the review, it is quite clear that mineral admixtures may be categorized into two groups, namely, chemically active mineral admixtures (highly reactive pozzolan) and microfiller mineral admixtures (low to moderate reactive pozzolan). *SF* and *MK* are chemically active mineral admixtures, whereas *FA*, *GGBS,* and *RHA* are microfiller mineral admixtures. Based on these two groups, the following generalized conclusions can be drawn on the properties of fresh concrete.

1. Chemically active mineral admixtures (highly reactive pozzolan) increase the cohesiveness of concrete and require more water to maintain workability; however, the requirement of water may be offset by adding plasticizer. The water demand depends on the particle size, specific surface area, particle shape, replacement level, and reactivity of particular mineral admixture.
2. The workability of concrete with microfiller mineral admixtures (low to moderate reactive pozzolan) greatly depends on the particle size, specific surface area, particle shape, and replacement level. In general, smaller the particle size and higher the specific surface of mineral admixture increases the water demands of concrete. The low to moderate reactivity and filler effect help to maintain the workability and sometimes increase the workability.
3. Heat of hydration increases with the use of chemically active mineral admixtures and decreases with the use of microfiller mineral admixtures.
4. Initial and final setting time of concrete depends on water content, initial and curing temperature, dosage of superplasticizer, and the reactivity of mineral admixture. Thus, on similar conditions, initial and final setting times of concrete decrease with the use of chemically active mineral admixtures and increase with the use of microfiller mineral admixtures.
5. In general, setting time of concrete decreases with the increase in replacement levels of chemically active mineral admixtures and increases with the increase of microfiller mineral admixtures.
6. All mineral admixtures studied reduce bleeding in concrete with correct proportion of all ingredients.
7. Based on Chapelle test, the reactivity of mineral admixtures is of the order: *MK* > *SF* > *FA* > *GGBS*.
8. Smaller particle size and higher specific surface area of mineral admixtures are favourable to produce highly dense and impermeable concrete; however, they cause low workability and more water demand which may be offset by adding effective superplasticizer.

## Figures and Tables

**Table 1:** Comparison of physical and chemical properties of OPC and mineral admixtures.

	OPC	*FA *	*GGBS *	SF**	*MK *	*RHA *	Remarks
Specific gravity	3.05 [48]	2.2–2.8 [28]	2.79 [48]	2.6–3.8	2.5	2.11 [44]	The specific gravity of mineral admixtures is generally lesser than cement. Therefore, more volume is obtained when mineral admixture replaces the cement.

SiO_2_, %	20.44	35–60* [28]	34.4 [48]	91.4	53.87	88.32 [44]	Amounts of SiO_2_ and Al_2_O_3_ are maximum in *SF* and *RHA* showing their reaction capability with the primary hydrate of cement
Al_2_O_3_, %	2.84	10–30* [28]	9.0 [48]	0.09	38.57	0.46 [44]	

Fe_2_O_3_, %	4.64	4–20* [28]	2.58 [48]	0.04	1.4	0.67 [44]	
CaO, %	67.73	1–35 [28]	44.8 [48]	0.93	0.04	0.67 [44]	
MgO, %	1.43	1.98 [28]	4.43 [48]	0.78	0.96	0.44 [44]	
SO_3_, %	2.20	0.35 [48]	2.26 [48]	0.01 [49]	—	—	
Na_2_O, %	0.02	0.48 [48]	0.62 [48]	0.39	0.04	0.12 [44]	
K_2_O, %	0.26	0.4 [48]	0.5 [48]	2.41	2.68	2.91 [44]	
MnO, %	0.16	—	—	0.05	0.01	—	
TiO_2_, %	0.17	—	—	0.0	0.95	—	

Particle size***, *μ*m	10–40 [16]	≤45 [28]	—	0.1 [42]	0.5–20 [50]	11.5–31.3 [44]	Smaller particle size within concrete causes a greater surface area to react more effectively with the alkaline environment [3].

Specific surface (m^2^/g)***	1.75 BET surface area	5–9 BET surface area [51]	0.4–0.599 BET surface area [48, 52]	16.455 BET surface area	12.174 BET surface area	30.4–27.4 [44] BET surface area	*RHA*,* SF*, and* MK *have the highest specific surface area. This results in highly dense and impermeable concrete with *RHA*, *SF*, and *MK*. The BET surface area is affected by the carbon content of the *SF *and* FA* (higher carbon, higher surface area) [42].

Loss on ignition, %	1.8	0.3–3 [28]	1.32 [48]	2.0	1.85	5.81 [44]	Higher carbon content results in higher LOI in mineral admixtures [42].

Pozzolan reactivity	—	0.875 [53]	0.040 [53]	1.288	1.342	—	Pozzolan reactivity (gm of Ca(OH)_2_ consumed per gm of pozzolan) chapelle test [53].

*If the sum of SiO_2_ and Al_2_O_3_ and Fe_2_O_3 _exceeds 70%, then it will be classified as ASTM C618 Class F *FA*; however, if the sum exceeds 50%, then it will be classified as ASTM C618 Class C *FA* [28]. The CaO content is generally higher than 20% in Class C *FA* [28].

**The chemical composition of *SF* varies with the type of alloy that is being produced [42].

***Particle size and specific surface of mineral admixtures vary and greatly depend on grinding mill and duration of grinding.
